# SIRT1 upregulation promotes epithelial-mesenchymal transition by inducing senescence escape in endometriosis

**DOI:** 10.1038/s41598-022-16629-x

**Published:** 2022-07-19

**Authors:** Minghua Wang, Yongqi Wu, Yunbiao He, Jing Liu, Yingxing Chen, Jieqiong Huang, Guolong Qi, Ping Li

**Affiliations:** 1 Department of Pathology, Longgang District People’s Hospital, Shenzhen, 518172 China; 2 Department of Pathology, Jinan University School of Medicine, Guangzhou, 510632 China; 3 Department of Medical Statistics, Jinan University School of Medicine, Guangzhou, 510632 China; 4 Department of Gynecology and Obstetrics, First Affiliated Hospital of Jinan University, Guangzhou, 510630 China

**Keywords:** Medical research, Cell biology, Cell migration, Cell signalling, Senescence, Diseases, Endocrine reproductive disorders, Molecular biology, Transcriptomics

## Abstract

Endometrial epithelial cells carry distinct cancer-associated alterations that may be more susceptible to endometriosis. Mouse models have shown that overexpression of SIRT1 associated with oncogene activation contributes to the pathogenesis of endometriosis, but the underlying reason remains elusive. Here, we used integrated systems biology analysis and found that enrichment of endometrial stromal fibroblasts in endometriosis and their cellular abundance correlated negatively with epithelial cells in clinical specimens. Furthermore, endometrial epithelial cells were characterized by significant overexpression of SIRT1, which is involved in triggering the EMT switch by escaping damage or oncogene-induced induced senescence in clinical specimens and in vitro human cell line models. This observation supports that genetic and epigenetic incident favors endometrial epithelia cells escape from senescence and fuel EMT process in endometriosis, what could be overcome by downregulation of SIRT1.

## Introduction

Endometriosis is characterized by the growth of endometrial glands and stroma-like tissue outside of the uterine cavity. Although controversies remain regarding the genesis of endometriotic lesions^1,2^, it is most commonly accepted that the endometrium is the main reason and that some cellular or genomic incident must have occurred to cause endometriosis^1^. At endometriotic sites, the ectopic glands always histologically resemble uterine endometrial glands but show variations in different epithelial-to-stromal cell ratios to the extreme case of stromal endometriosis^3^.

One of the mechanisms suggested to participate in the onset of endometriosis is the epithelial-mesenchymal transition (EMT)^4,5^. EMT is a process by which epithelial cells convert to motile mesenchymal cells. This mostly occurs during embryonic development and cancer and has been found in endometrial epithelial cells in vitro and in human endometrial tissues^6^. Once the ectopic endometriotic lesion is established, multiple mechanisms involved with EMT are activated, resembling cancer-like progression and invasive growth^7^. It is also well known that EMT endows epithelial cells with migratory and invasive properties, similar to metastatic carcinoma cells^8^. Furthermore, the cancer-associated mutations and/or activation are thought to have metastatic potential, are present in endometriosis and appear to be confined to the epithelial but not the stromal compartment^9^.

The human endometrium is a highly regenerative tissue undergoing growth and shedding throughout the menstrual cycle; both the senescence program and cancer-associated mutations are involved in endometrial regeneration^9,10^, and when perturbed, they can have detrimental effects during tumour progression and ageing^11,12^. Meanwhile, abnormal levels of cellular senescence in endometrial epithelial cells may be one of the causes of reproductive diseases, including miscarriage and endometriosis^13,14^. Moreover, it has been shown that the acquisition of EMT may be accompanied by the bypass of senescence in most cancers^15–17^. Due to the invasive behaviour and ageing phenotypes, we reasoned that escape from senescence of endometrial epithelial cells might contribute to the acquisition of metastatic features in endometriosis.

Sirtuin family proteins are class Ш histone deacetylases (HDACs) and the best known is the well-described role of SIRT1 in cancer and ageing^18^. Thus, we hypothesized that escape from senescence mediated by SIRT1 is associated with the epithelial to mesenchymal transition in endometriosis.

## Results

### Bioinformatic analysis showed that EMT and senescence are involved in endometriosis

**Figure 1:**
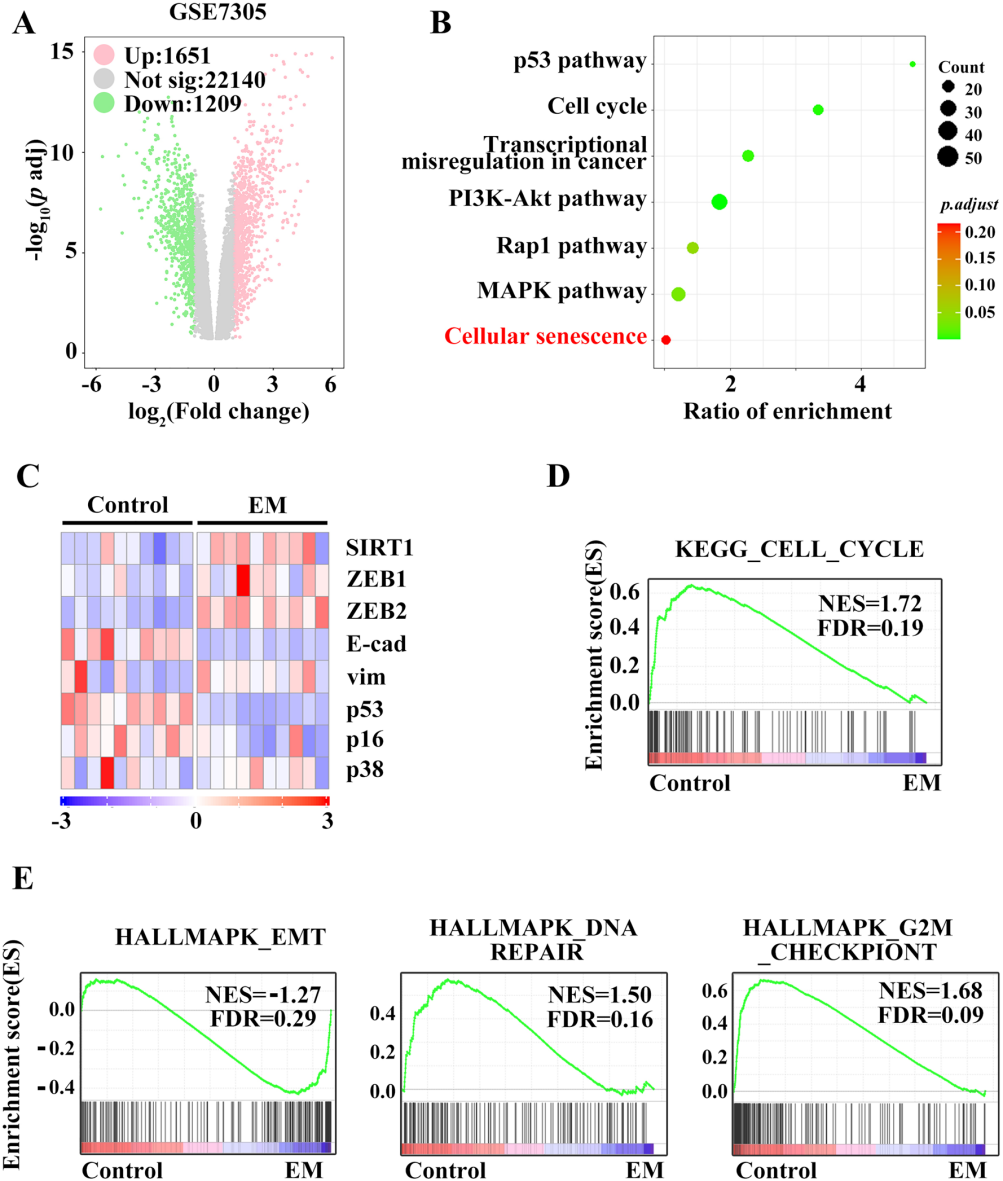
Bioinformatic analysis results support that EMT and senescence are involved in endometriosis. Differentially expressed genes (DEGs) were identified. The red and green indicate unregulated and downregulated DEGs, respectively, in GSE7305 (**A**). Hallmark pathway enrichment (**B**) and heatmap of EMT- and senescence-related genes from GSE7305 (**C**). Endometriosis showing enhanced genes in the “cell cycle” pathway (**D**). GSEA revealed enrichment of endometriosis genes in “EMT”, “DNA repair” and “G2/M checkpoint” (**E**).

To gain insights into the pathological changes during endometriosis, we analysed differentially expressed genes (DEGs) in the public dataset GSE7305, GSE11691 GSE5108 and E-MEXP-1251, which contains microarray data of endometriotic and normal endometrial specimens (Fig. 1A and Supplementary Figs. S1–S3). Pathway enrichment analysis identified significant dysregulation of the previously described crosstalk between senescence and EMT pathways^19,20^, such as “p53”, “MAPK” and “cell cycle” (Fig. 1B). Furthermore, GSEA was performed to explorer differences in endometriosis and and its related signal pathway for senescence and EMT (Fig. 1C). KEGG enrichment analysis showed that endometriosis was associated with the cell cycle (Fig. 1D).The HALLMARK gene sets showed positive enrichment of genes in EMT and positive enrichment of genes in cell cycle arrest, including the cell cycle, G2/M checkpoint and DNA repair (Fig. 1E), which are known to be linked to cellular senescence.

**Figure 2:**
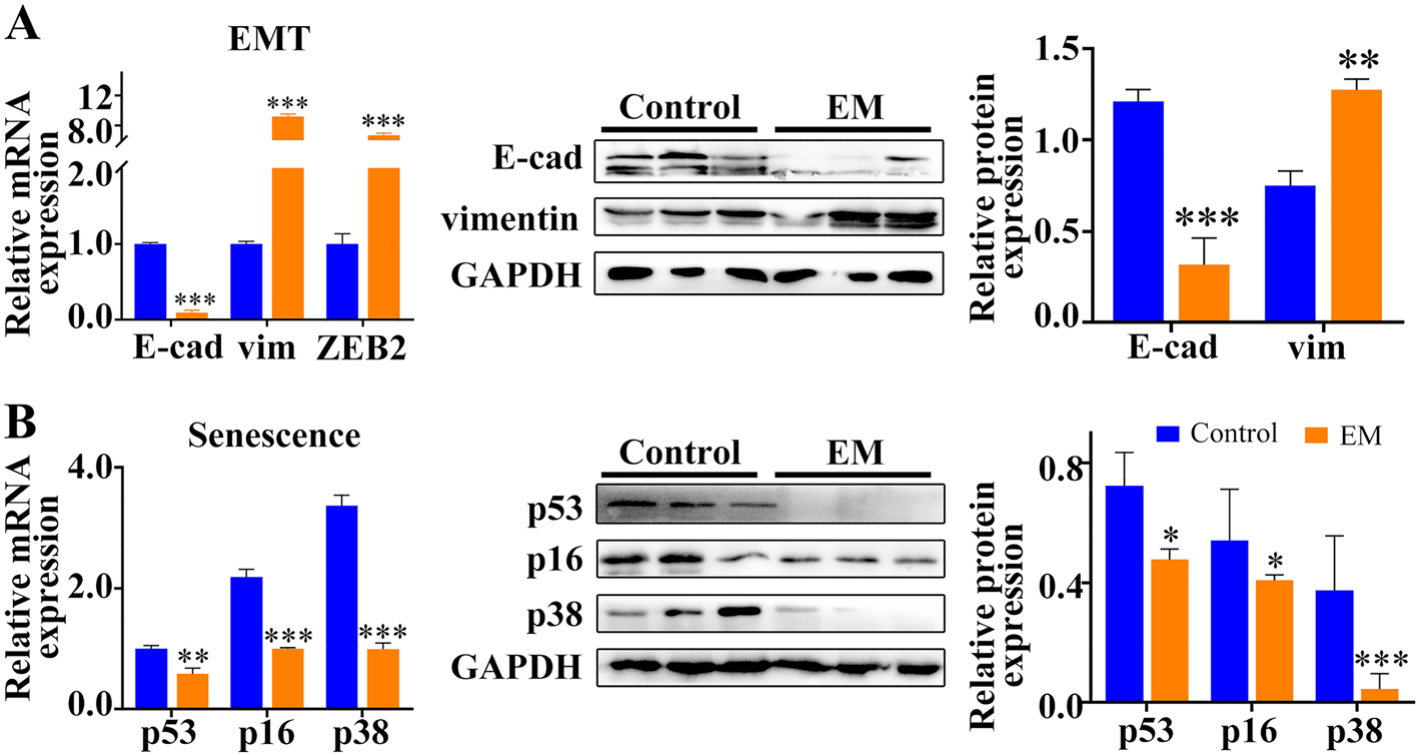
Relative expression of EMT- and senescence-related genes Expression as shown by qRT-PCR and western blotting, respectively (**A**,**B**). Anti-GAPDH antibody was used as loading control. Greyscale values of the bands were determined by Quantity One software. *p < 0.05, **p < 0.01, ***p < 0.001.

We then examined the expression levels of EMT- and senescence-related genes in clinical endometriotic specimens and found that both the mRNA and protein levels of EMT-related genes (E-cadherin, vimentin and ZEB2) were abnormally expressed in ectopic endometrial tissues (Fig. 2A, n = 10). In contrast, senescence-related gene levels (p53, p16 and p38 MAPK) were suppressed (Fig. 2B, n = 10).

### Senescence and EMT occur in epithelial cells of of endometriosis

**Figure 3:**
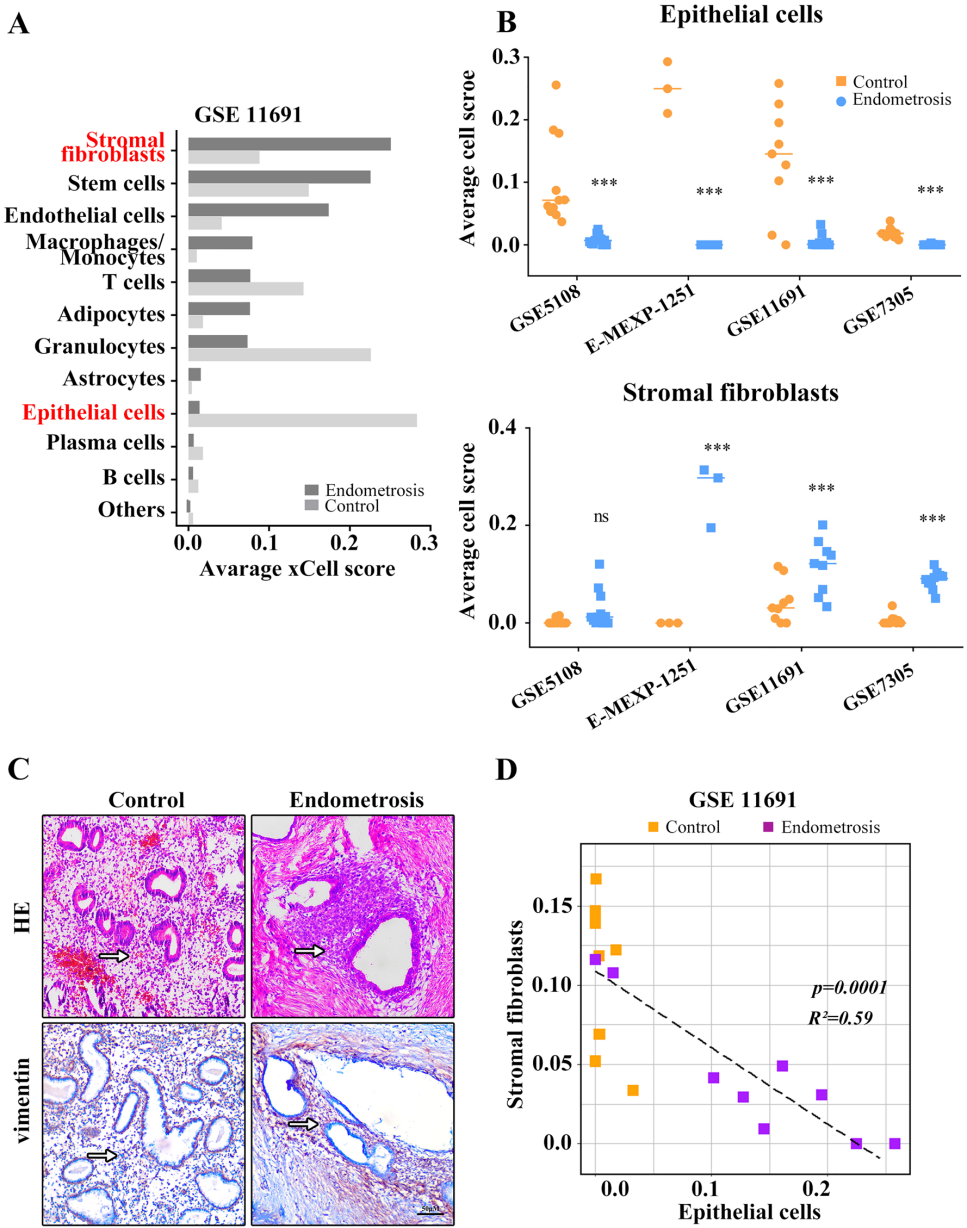
Endometrial stromal fibroblasts were enriched in endometriosis compared to other cell types. Computational biology analysis revealed the total content of different cells in the tissues of the control and endometriosis (GSE11691, n = 10) (**A**). Endometrial stromal fibroblast and epithelial cell abundance scores in tissues of the control and endometriosis, as calculated by xCell (**B**). HE staining and vimentin immunohistological staining showing stromal fibroblasts and epithelial cells (**C**). Abundance score correlation analysis for stromal fibroblasts with epithelial cells in tissues of the control and endometriosis (**D**). Scale bars = 50 µm in (**C**). ***p < 0.001.

Since the presence of numerous cell types within samples can influence gene signatures, we applied the xCell package to determine the tissue cellular heterogeneity of endometriosis in these four datasets (Fig. 3 and Supplementary Fig. S4). In GSE11691, the most prevalent cells were fibroblasts, stem cells and endothelial cells, and very few epithelial cells were found in endometriosis (Fig. 3A). We then compared the frequency of each cell type between endometriotic and normal endometrium, which indicated that there was a significantly different composition of the cell types between endometriosis and control, and fibroblasts were enriched (the most abundant) cell type in endometriotic endometrium, while epithelial cells were enriched (the most abundant) cell type in control endometrium (Fig. 3B). For external validation, we confirmed a decrease in epithelial cells and an increase in fibroblast abundance in endometriosis compared with control tissues in both GSE7305 and GSE5108, as well as in endometrial tissues (Fig. 3B,C). The number of epithelial cells negatively correlated with the number of fibroblasts in tissues of the control and endometriosis in GSE11691 (Fig. 3D). The full list of scores for all specific cell types is presented in the Supplementary Table S1. Similar results were also noted in other three dataset (Supplementary Fig. S5). Overall, the fact that epithelial cells can transform into stromal cells supports that they may contribute to the high fibroblast abundance and the pathogenesis of endometriosis.

**Figure 4:**
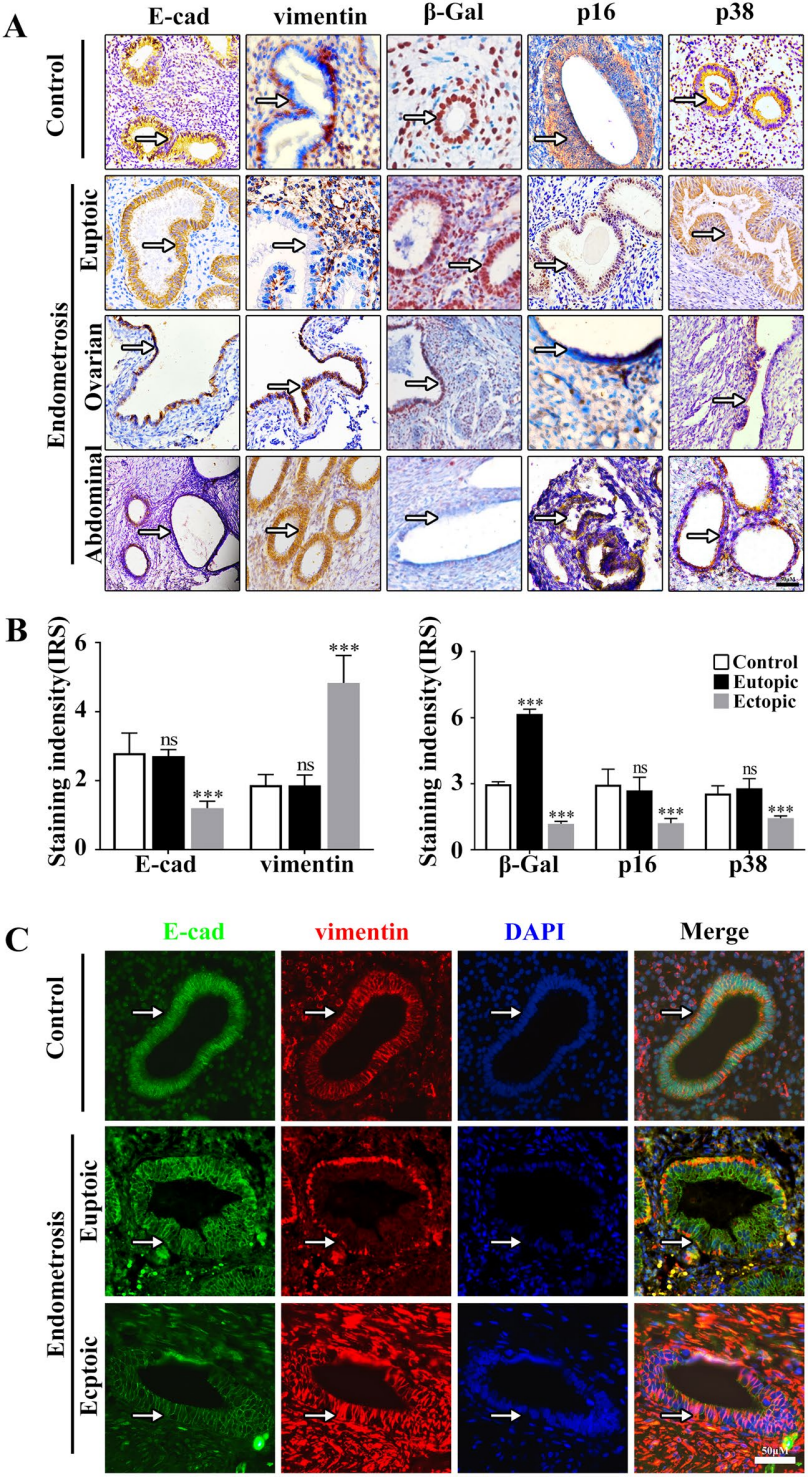
EMT- and senescence-related protein expression in epithelial cells of endometriosis. Immunostaining for E-cad, vimentin, β-gal, p16 and p38 (**A**). The intensity of the immunohistological staining was given an IRS score as the mean ± SEM. The results show that E-cad and vimentin expression was stronger, but β-gal, p16 and p38 expression was weaker in epithelial cells in endometriosis tissues than in the controls (**B**). E-cad and vimentin protein levels were examined by immunofluorescence staining (**C**). Scale bars = 50 µm in (**A**,**C**). ***p < 0.001.

Although the impact of aging on uterine function is less clear^21^, it has been demonstrated that endometrial epithelial cells display characteristic features of senescence in cyclic endometrium^22^, whereas in endometrial cancer, cell escapes from senescence and acquires the aggressive phenotype^23^. Based on these observations, we evaluated senescence and EMT markers using immuhistochemistry in endometrial specimens. Consistent with the previous studies^6^, epithelial cells in ectopic lesions showed a decrease of E- cadhein (a hallmark of EMT) and an increase of vimentin (mesenchymal marker). In contrast, the expression of senescence markers including β-gal, p16 and p38 MAPK were decreased in ectopic lesions compared to normal and eutopic endometriotic lesions (Fig. 4A–C).

### Both the previous literature and human samples highlight the correlation between SIRT1 upregulation and endometriosis

**Figure 5:**
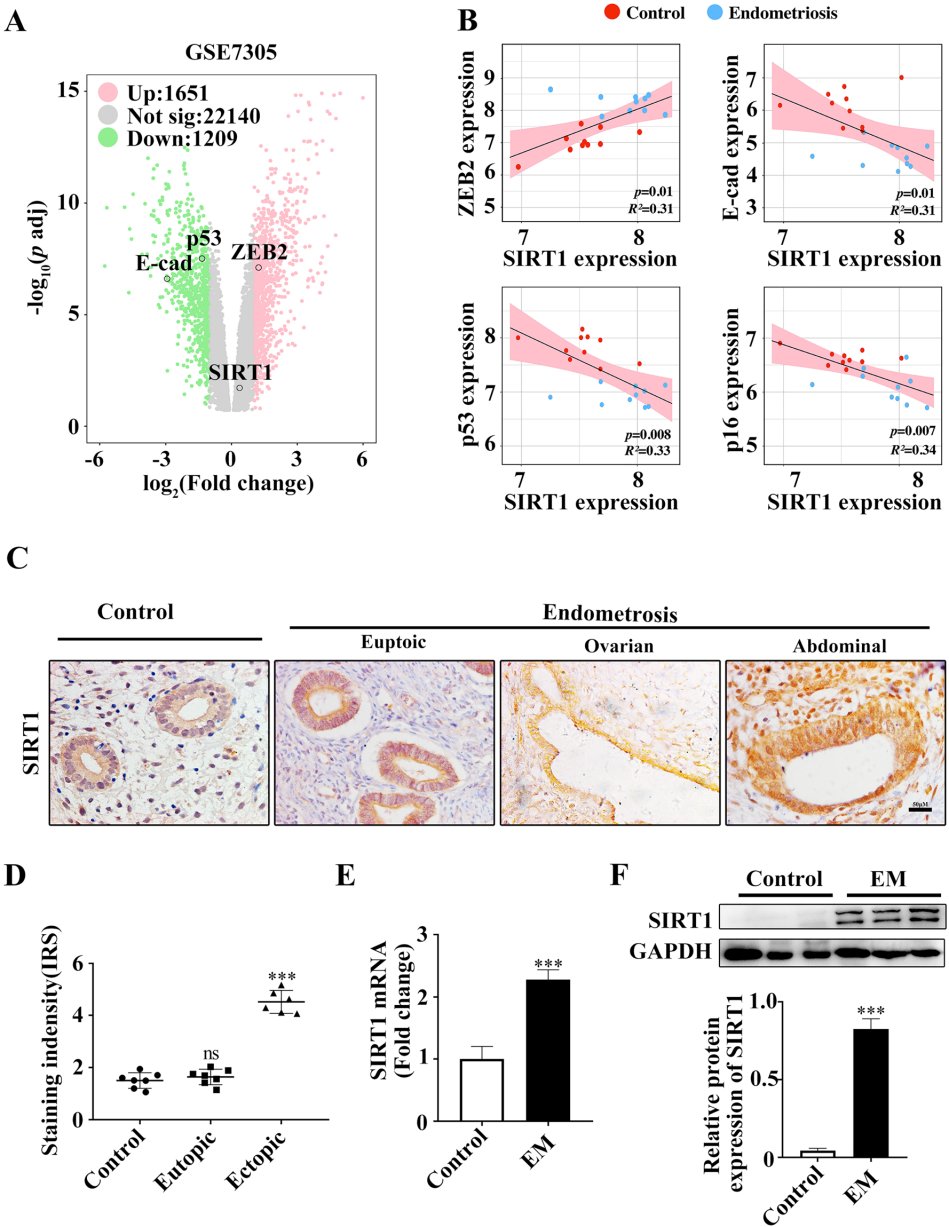
The expression of SIRT1 was unregulated in endometriosis. A volcano plot shows the high expression of SIRT1 and ZEB2 and the low expression of E-cad and p53 in endometriosis (**A**). Correlation analysis of SIRT1 with ZEB2, E-cad, p53 and p16 in endometriosis (**B**). Representative images of SIRT1 expression were detected by IHC in control and endometriotic endometria (**C**). Dot plot of the IRS scores. The differences were analysed by Student’s t-test. ***p < 0.001 (**D**). qPCR (**E**) and western blots (**F**) were used to detect the SIRT1 expression levels in the control and endometriotic endometrium. *E-cad* E-cadherin, *EM* endometriosis. Scale bars = 50 µm in (**C**).

Having demonstrated the decreased cellular senescence and enhanced EMT in endometriosis, we then aimed to investigate the molecular mediators of the process. Although previous literature^7,24–42^ demonstrated that EMT and endometriosis are closely associated with SIRT1 genes, indicating the involvement of SIRT1 in the pathogenesis of endometriosis (Table 1), no study has been performed to explore whether SIRT1 regulates EMT and senescence in endometriosis. We thus examined the effect of SIRT1 on the associated differential gene expression. In dataset GSE7305, the volcano plot heighted that SIRT1, ZEB_2_, p53 and E-caherin were the significantly differentially expressed between the endometriosis group and the control group (Fig. 5A). SIRT1 and ZEB_2_ were differentially expressed with significantly higher expression in endometriotic specimens (FC = 1.29, *p* = 0.005; FC = 0.83, *p* < 0.001, respectively), while E-cadherin and p53 showed significantly lower expression in endometriotic specimens (FC = 0.13, *p* = 0.016; FC = 0.39, *p* < 0.001, respectively) (Fig. 5A). Correlation analysis was used to determine the degree of association. SIRT1 expression was positively correlated with ZEB_2_ (*p* = 0.01, *R*^*2*^ = 0.31), while SIRT1 expression was inversely correlated with E-cadherin (*p* = 0.05, *R*^*2*^ = 0.18), p53 expression (*p* = 0.008, *R*^*2*^ = 0.33), and p16 expression (*p* = 0.007, *R*^*2*^ = 0.34) (Fig. 5B), indicating the underlying regulatory mechanism. Similar results were also observed in the GSE11691, GSE5108 and E-MEXP-1251 dataset (Supplementary Fig. S6).

**Table 1:** The literature has demonstrated that EMT and endometriosis are closely associated with SIRT1 genes.

	Study	Endometriosis		Serum	Endometriosis		Endometriosis and EMT
		Human	Mouse		ectopic	eutopic	
1	Taguchi et al. (2014)	✓			✓	✓	
2	Asaka et al. (2015)	✓	✓		✓	✓	
3	Proestling et al. (2015)	✓			✓	✓	✓
4	Yoo et al. (2015)	✓	✓	✓	✓	✓	
5	Zhang et al. (2017)	✓	✓		✓	✓	✓
6	Chatterjee et al. (2018)	✓	✓	✓	✓	✓	✓
7	Xiong et al. (2019)	✓			✓	✓	✓
8	Chiang et al. (2020)	✓	✓	✓	✓	✓	✓
9	Kong et al. (2020)	✓	✓		✓	✓	✓
10	Teasley et al. (2022)	✓			✓	✓	
11	Zhang et al. (2020)	✓			✓	✓	✓
12	Zheng et al. (2020)	✓			✓	✓	✓
13	Cela et al. (2021)	✓		✓	✓		✓
14	Chen et al. (2021)	✓			✓		
15	Khodarahmian et al. (2021)	✓			✓		
16	Kim et al. (2021)	✓	✓		✓	✓	
17	Rezk et al. (2021)	✓		✓	✓	✓	
18	Sansone et al. (2021)	✓		✓	✓	✓	
19	Wang et al. (2021)	✓	✓	✓	✓	✓	
20	Wei et al. (2021)	✓	✓		✓	✓	✓

The expression of SIRT1 was investigated in endometriosis and control tissues by IHC. SIRT1 protein was expressed in both endometrial glandular and stromal cells. In the epithelial cells, SIRT1 level was increased in both the eutopic and ectopic endometrium of endometriotic patients, compared with normal endometrium (*p* < 0.0001, for both), but its levels in the eutopic and ectopic endometrium of endometriotic patients did not differ (*p* = 0.67) (Fig. 5C,D). Accordingly, the PCR and western blot results showed that SIRT1 expression was upregulated in ectopic endometrial tissues compared with normal control endometrial tissues (Fig. 5E,F).

### Overexpression of SIRT1 allows endometrial epithelial cells (EECs) to evade senescence

**Figure 6:**
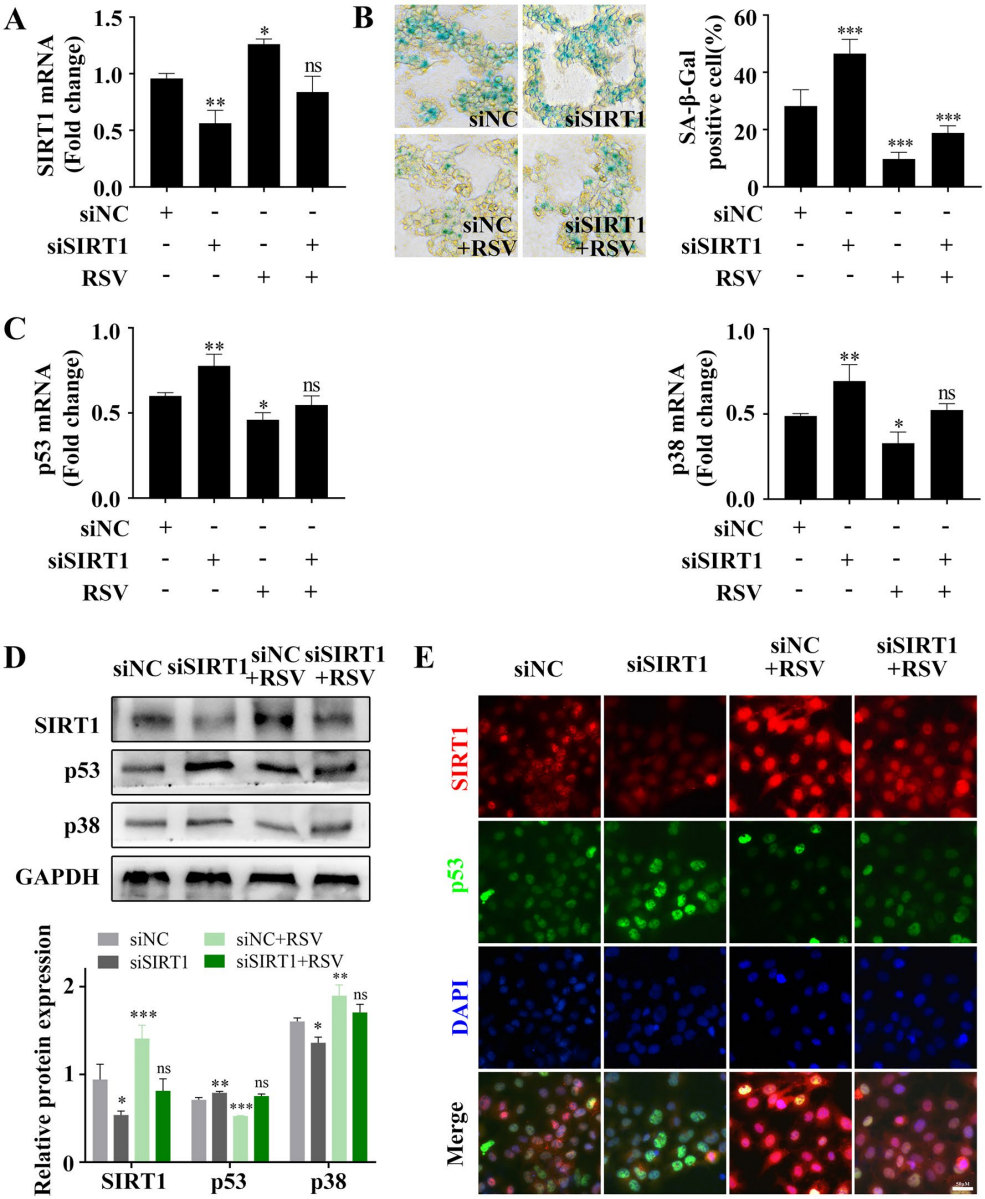
Effect of RSV on the senescence of Ishikawa cells**.** qPCR analysis of SIRT1 mRNA levels in Ishikawa cells first transfected with siNC or siSIRT1 and then treated with or without RSV for 24 h (**A**). Representative SaβG staining in parallel Ishikawa cell cultures (**B**). qPCR analysis of p53 and p38 mRNA levels in Ishikawa cells first transfected with siNC or siSIRT1 and then treated with or without RSV for 24 h (**C**). Representative western blot and quantification of senescence-related genes from parallel cell cultures (**D**). p53 and SIRT1 protein levels were examined by immunofluorescence staining (**E**). *RSV* resveratrol. Scale bars = 50 µm in (**E**). *p < 0.05, **p < 0.01, ***p < 0.001.

To confirm the activity of SIRT1 in endometrial epithelial cells, resveratrol (RSV) as a SIRT1 activator and siRNA targeting SIRT1 were used in the Ishikawa cell line. qRT–PCR analysis showed that SIRT1 mRNA content was reduced by 49% and that RSV reversed the downregulation of SIRT1 in response to siRNA (Fig. 6A). RSV restored whereas knockdown of SIRT1 promoted cellular senescence in Ishikawa cells as ascertained by SAβG (Fig. 6B). As expected, SIRT1 knockdown was associated with an increase in the expression of p53 and p38MAPK, and RSV abolished the upregulation of senescence-associated genes in Ishikawa cells (Fig. 6C,D). Furthermore, immunofluorescence staining showed that SIRT1 knockdown induced p53 accumulation and downregulation of p53 in response to RSV (Fig. 6E). The data indicate that the upregulation of SIRT1 induced by RSV is SIRT1- dependent and allows cells to escape senescence.

### Overexpression of SIRT1 promotes epithelial cells (EECs) EMT

**Figure 7:**
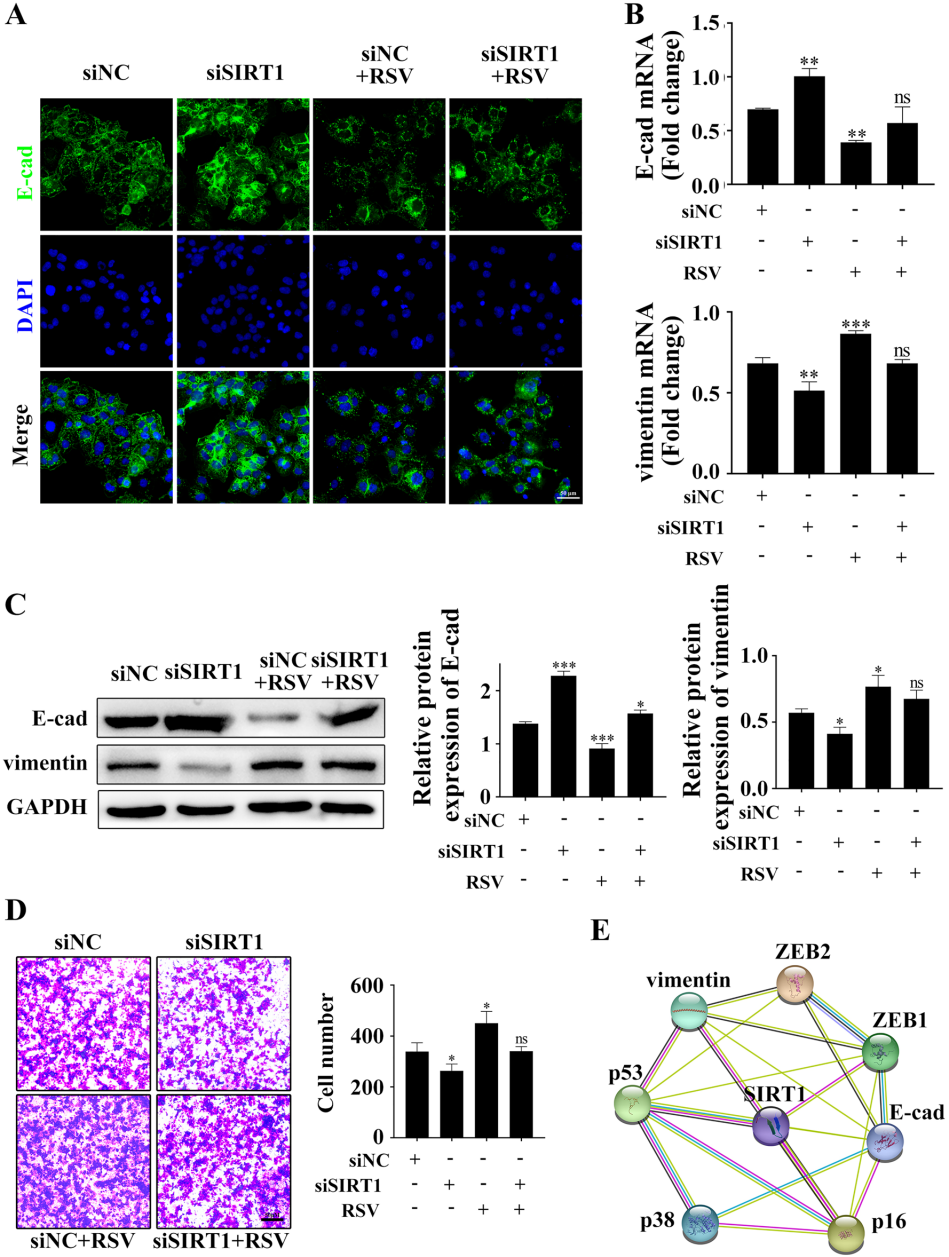
Effect of RSV on the EMT of Ishikawa cells**.** Ishikawa cells were treated with siNC or siSIRT1 with or without RSV, and then E-cad protein levels were examined by immunofluorescence staining (**A**). E-cad and vimentin mRNA or protein levels were examined by qPCR (**B**) and western blotting (**C**). Matrigel invasion assays to determine the invasive properties of the cells from the parallel cell cultures (**D**). The bioinformatics analysis depicting the correlation between SIRT1, ECM and senescence was mapped with the STRING database to construct a PPI network (**E**). Scale bars = 50 µm in (**A**). *RSV* resveratrol, *E-cad* E-cadherin. *p < 0.05, **p < 0.01, ***p < 0.001.

Since SIRT1 has been shown to promote EMT and favour the metastatic process in endometrial cancer^24^, we next examined the features of EMT in the activation or knockdown of SIRT1 in Ishikawa cells. Consistent with SIRT1 knockdown-mediated cellular senescence, a significant increase in the E-cadherin epithelial marker and a decrease in the vimentin mesenchymal marker were observed in Ishikawa cells. In contrast, RSV triggered a partial loss of E-cadherin, an increase in vimentin expression (Fig. 7A–C) and the acquisition of migration and invasive properties (Fig. 7D and Supplementary Fig. S7). The regulation of SIRT1 signalling through the interplay between senescence and EMT in endometrial epithelial cells is mapped in Fig. 7E. Together, these data suggest that overexpression of SIRT1 allows Ishikawa cells to override cancer-associated senescence and promote EMT, thereby facilitating endometriosis migration and invasion.

## Discussion

In the current study we demonstrated that two important processes, senescence and EMT are involved in endometriosis. In endometrial epithelial cells, SIRT1 overrides the senescence program and contributes to the induction of EMT and the acquisition of metastatic capacity.

EMT represents a biological program during which epithelial cells lose their cellular identity and acquire a mesenchymal phenotype. This process is associated with the acquisition of tissue invasiveness. Epithelial-mesenchymal plasticity promotes the spread of ectopic lesions and can be activated by a number of signaling pathway in endometriosis^33,43^. Multiple cancer-related genes are believed to be EMT drivers. However, while it has been postulated that the endometrial epithelial cells carried distinct cancer-associated alterations that may be more susceptible to endometriosis^44,45^, it is still an open question of how EMT is triggered during endometriosis. Furthermore, the intimate crosstalk between EMT and the failsafe program has been confirmed^16^, but it is not clear whether SIRT1 regulates EMT, senescence and the capacity of metastasis in endometrial epithelial cells. Accordingly, its upregulated expression is associated with the pathogenesis of endometriosis.

Recent studies have shown that senescent cells are present in different types of tissues, including the human endometrium^22^. Consistent with our current study, it could be suggested that the senescent endometrial epithelial cells share features of damage-induced senescence, including the increased SAβG activity and the expression of senescence-related markers (p53, p16 and p38MAPK)^22^. In addition, telomere shortening has been established as one of the major mechanisms of aging^46^. Ectopic endometriotic lesions have aberrant telomerase and longer telomeres in the epithelial cells^47^, suggesting that epithelial cells may escape senescence in endometriosis. Indeed, several signalling pathways have been found to be altered during trancriptome analysis of endometria from endometriosis. Among these are the p53, RAS (rat sarcoma virus) and MAPK-dependent pathways^4^. The role of SIRT1 in endometriosis was previously demonstrated in mice and humans, which suggests that an increase in SIRT1 expression is associated with oncogene KRAS activation and contributes to progesterone resistance in endometriosis^27,48^. Importantly, SIRT1 is an essential factor that delays cellular senescence by regulating diverse biological processes by deacetylating transcription factors, including p53, forkhead transcription factors (FOXOs), p38MAPK and SMADs^49^. Therefore, endometrial epithelial cells retain the potential to escape senescence by mechanisms that involve ectopic SIRT1 overexpression in endometriosis. Our results showed that SIRT1 was overexpressed in endometriosis and that its induction is sufficient to override damage induced senescence in epithelial cells by inhibiting both the p53 and p38MAPK pathways. It has been KRAS can activate senescence-like growth arrest, whereas SIRT1 overexpression inhibits KRAS-induced senescence and SIRT1 knowdown reduces ability of SIRT1 to prevent KRAS-induced senescence^50^, which was consistent with our results. Although it has been suggested that pharmacological inhibition of SIRT1 induces senescence or apoptosis, it is difficult to rule out off-target effects due to the limited specificity of these compounds^51^. Therefore, our hypothesis was further verified by the overexpression and knockdown of SIRT1. We demonstrated that, the inverse correlation between p53, p38 MAPK and SIRT1 protein in the endometrium also supports the role of SIRT1 protein as a negative modulator of p53 and p38 MAPK, as a crucial inhibitor of damage-induced senescence in human endometrial epithelial cells. One of the limitations is that we employed Ishikawa human endometrial adenocarcinoma cell line as a model of endometrial epithelial cells. Using immortalized endometrotic epithelial cell lines may provide a more convincing model for studying endometriosis in the future. SIRT1 is implicated in EMT activation, and its function in DNA repair and cell cycle control was found to be similar to that of a tumor promoter gene^50,51^. The best characterized event occurring in EMT is loss of E-cadherin. SIRT1 can deacetylate histone H3 and cause repression of E-cadherin^50^. Furthermore, a high frequency of SIRT1 overexpression was reported during tumor progression in endometrial carcinoma^24,52^. Noticeably, endometriosis shares many features with malignancy, such as EMT activation^33^. Indeed, activation of EMT is linked to the suppression of cellular senescence and escape from senescence is a general prerequisite for malignant conversion^46^. Further characterization at the molecular level showed that alterations in the p53- and p38MAPK-dependent pathway promote EMT^53^. Alternatively, the EMT in endometriotic epithelial cells might be driven by ZEB (zinc finger E-box binding)^40,54^. ZEB1 is required for SIRT1 recruitment to repress E-cadherin and EMT^51^. Downregulation of ZEB1 or ZEB2 triggers senescence both in vivo and in vitro via p53 or p21 activation^19,55,56^. Moreover, ZEB1 and ZEB2 expression are associated with SIRT1 overexpression and have been implicated in the suppression of p53 and p38 MAP^49,57^. Thus, EMT-competent cells may be capable to negating senescence checkpoint functions through ZEB1 and ZEB2, whereas cellular senescence may prevent SIRT1 from inducing ZEB to activate the EMT program.

In conclusion, we used integrated systemic biology analysis and found that enrichment of stromal fibroblasts and the potential activation of EMT are features of endometriosis tissues. Furthermore, the endometriotic lesions are characterized by significant overexpression of SIRT1 in endometrial epithelial cells, which is involved in triggering the EMT switch by escaping damage or senescence during progression of the pathology. One the limitations in endometrosis is This observation supports that genetic and epigenetic incident favors endometrial epithelia cells escape from senescence and fuel EMT process in endometriosis, what could be overcome by downregulation of SIRT1. Although further work is warranted in clarifying the off-target effects^51^, the development of SIRT1 modulators with enhanced potency and selectivity will be useful for treating endometriosis patients.

## Methods

### Public datasets

We used “endometrosis” as a keyword in the Gene Expression Omnibus (GEO) database, and four mRNA transcription datasets were collected, including GSE11691 (n = 9), GSE7305 (n = 10), GSE5108, (n = 11), and E-MEXP-1251(n = 12). The raw data were downloaded from the corresponding databases (https://www.ncbi.nlm.nih.gov/geo, and https://www.ebi.ac.uk/arrayexpress/) and analysed using the limma package (version 3.12) in R software (version 3.61), and genes with *p* value < 0.05 and Log_2_ (fold change) > 1 were considered DEGs.

### Data analysis

Gene Ontology (GO) and KEGG pathway enrichment analysis for DEGs were performed using the “clusterProfiler” package (version: 3.13) of R (http://bioconductor.org/packages/release/bioc/html/clusterProfiler.html). KEGG and HALLMARK gene sets were used from the Molecular Signatures Database (MSigDB). Gene set enrichment analysis (GSEA) was implemented by version 4.1.0, and the enrichment results were interpreted by normalized enrichment score^9^ and a false discovery rate (FDR) value < 0.05.

The protein–protein interaction network of the chosen genes was constructed by STRING (https://string-db.org, version 11.5), with a score > 0.400 corresponding to a median confidence interaction as significant.

To dissect the abundance of cell types in endometrial tissues, the xCell package (version: 1.36.0) (https://github.com/dviraran/xCell) was used to explore the tissue cellular heterogeneity from the gene expression data.

### Clinical sample collection

This study was performed in accordance with the ethical principles outlined by the Helsinki Declaration and was approved by the Clinic Trial Ethics Committee of Longgang District People’s Hospital, Shenzhen, China (approval NO. 2022012). After informed consent, endometriosis subjects were recruited from women presenting with symptoms and subsequently confirmed by laparoscopic surgery and postoperative histological examination. Staging was performed according the revised American Fertility Society (r-AFS) classification into minimal-mild (stage I/II) and moderate-severe (III/IV) groups. Healthy fertile subjects were recruited from patients undergoing interval tubectomy and classified as controls. All subjects with age ≥ 40 years, ovarian tumors, polycystic ovarian syndrome, anatomical uterine abnormalities, pelvic inflammatory diseases and other pelvic pathological conditions were excluded. Additionally, all involved subjects had not received any hormonal treatment and did not use an intrauterine contraceptive device in the last 3 months prior to examination and sampling. Blood and tissue samples were collected during the proliferative phase of the menstrual cycle, which was determined by preoperative history and histological examination. The patient details are shown in Table 2.

**Table 2:** Clinical characteristics of samples included in the study.

		Controls (n = 14)	Endometriosis (n = 15)
Age(years), Mean ± SD		37.4 ± 7.5	32.7 ± 7.6
Diameter (cm)	< 3	−	3
	> 3	−	12
Type	Ovarian endometriosis	−	8
	Abdominal wall endometriosis	−	7
	Ovarian & Abdominal wall endometriosis	−	10
r-AFS stage	I /II	−	5
	III /IV	−	10
Symptoms	Dysmenorrhea	−	3
	Abnormal menstruation	−	6
	Chronic pelvic pain	−	6
CA125(U/ml), Mean ± SD		24.2 ± 7.7	79.9 ± 68.5

*r-AFS* revised American Fertility Society.

### Immunohistochemistry and immunofluorescence microscopy

Tissues were fixed, embedded in paraffin, sectioned (5 µm thick) and stained with haematoxylin and eosin (H&E) and Masson’s trichrome. The methods was performed as previously described^57^. Antibodies were used against the following targets: β-galactosidase (β-gal) (1:100 dilution, Santa Cruz, USA), p16 (1:100 dilution, CST, USA), p38 (1:50 dilution, Abcam, USA), E-cadherin (1:100 dilution, BD Biosciences, USA), vimentin (1:100 dilution, CST, USA) and SIRT1 (1:200 dilution, Abcam, USA). For the negative control, the primary antibody was replaced with an isotype control antibody. Immunohistochemistry and immunofluorescent staining and semiquantitative analysis by immunohistochemical reactive scores (IRSs) were conducted as previously described. Photographs were taken using an Olympus IX51epi-fluorescence microscope.

### RT-qPCR and western blotting analysis

RT–qPCR and western blotting were performed as previously described^57^. The specific primers and antibodies are presented in Supplementary Table S2. The expression of genes was normalized to GAPDH. The results are representative of three independent experiments.

### Cell culture and RNA interference

Ishikawa cells (BNCC, China) were cultured in DMEM/F12 (HyClone, USA) supplemented with 5% fetal bovine serum (FBS) (5% CO_2_, 20% O_2_, 37 °C). For the experiments, subconfluent Ishikawa cells were incubated in resveratrol (RSV) (Selleck, USA) for 24 h. The siRNAs against human SIRT1 (siSIRT1) and a negative control (siNC) were purchased from Guangzhou RiboBio Co., Ltd. (Guangzhou, China), and the sequences are presented in Supplementary Table S2. Cells were transfected with siRNA using Lipofectamine according to the manufacturer’s instructions (Invitrogen, USA).

### SAβG staining

Senescent cells were detected using the Senescence β-Galactosidase Staining Kit (Beyotime, China) and observed under a phase-contrast microscope^57^. The quantification of positive cells was performed by counting the cells in 5 random fields per dish. At least 300 cells were randomly counted from each sample.

### Transwell assay

A total of 5 × 10^4^ cells in 100 µL serum-free DMEM/F-12 were seeded into the upper compartment of a 24-well chamber precoated with Matrigel (BD Biosciences, USA). The lower chamber was filled with DMEM/F 12 containing 10% FBS. After 24 h of incubation, cells on the upper surface of the filter were removed by wiping with a wet cotton swab and it was washed several times with PBS. The migrated cells on the lower surface of the upper chamber were fixed with 4% paraformaldehyde, stained with 0.1% crystal violet, and counted in five random fields under a light microscope.

### Statistical analysis

GraphPad Prism software 8.0 was used to analyse the gene expression data generated from the IHC, qPCR and western blotting. Statistical significance was defined as *p* < 0.05. For single comparisons, unpaired t-tests were performed. One-way analysis of variance (ANOVA) was used for multiple comparisons. The results are presented as the mean ± SD for normally distributed data or as the median and interquartile range for nonnormally distributed data.

## Supplementary Information

Supplementary Information 1.

Supplementary Information 2.

Supplementary Information 3.

Supplementary Information 4.

Supplementary Information 5.

Supplementary Information 6.

Supplementary Information 7.

Supplementary Information 8.

Supplementary Information 9.

Supplementary Information 10.

Supplementary Information 11.

Supplementary Information 12.

Supplementary Information 13.

Supplementary Information 14.

Supplementary Information 15.

## Data Availability

The GSE11691, GSE7305 and GSE5108 datasets analyzed during the current study are available in the Gene Expression Omnibus data base, [https://www.ncbi.nlm.nih.gov/geo/]. The E-MEXP-1251 dataset analyzed during the current study are available in the Array Express, [https://www.ebi.ac.uk/arrayexpress/experiments/E-MEXP-1251/].
