# Supplementary material for: SIRT1 upregulation promotes epithelial-mesenchymal transition by inducing senescence escape in endometriosis
Source: Sci Rep. 2022 Jul 19;12:12302. doi: 10.1038/s41598-022-16629-x (PMC9296487; doi:10.1038/s41598-022-16629-x)
Supplement: Supplementary file 1 — Supplementary Information 1. [file 41598_2022_16629_MOESM1_ESM.docx]

**Supplementary Figure Legends**

**Supplementary Fig. S1.** Bioinformatic analysis in GSE11691 dataset.

**Supplementary Fig. S2.** Bioinformatic analysis in GSE5108 dataset.

**Supplementary Fig. S3.** Bioinformatic analysis in E-MEXP-1251 dataset.

**Supplementary Fig. S4.** Computational biology analysis of the total content of different cells in the tissues of the control and endometriosis (GSE 7305, GSE5108 and E-MEXP-1251).

**Supplementary Fig. S5.** Abundance score correlation analysis for stromal fibroblasts with epithelial cells in tissues of the control and endometriosis (GSE7305, GSE5108 and E-MEXP-1251).

**Supplementary Fig. S6.** Expression and correlation analysis of SIRT1 with ZEB2, E-cad, p53 and p16 in endometriosis in GSE11691, GSE5108 and E-MEXP-1251.

**Supplementary Fig S7.** Wound healing assays in Ishikawa cells transfected SIRT1 siRNA and /or treated RSV, respectively.

**Supplementary Fig S8.** Original blot images files in Fig2.

**Supplementary Fig S9.** Original blot images files in Fig5.

**Supplementary Fig S10.** Original blot images files in Fig6.

**Supplementary Fig S11.** Original blot images files in Fig7.

**Supplementary Table S1**. Cell type enrichment score obtained by transcriptome analysis from eutopic endometrium samples from healthy and women with endometriosis.

**Supplementary Table S2** Gene expression by transcriptome analysis from eutopic endometrium samples from healthy and women with endometriosis was used for correlation analysis in GSE7305.

**Supplementary Table S3** Primer sets used for RT-PCR, and sequence of siRNAs used for small interference RNA experiments.
