# Supplementary figures and images for: SIRT1 upregulation promotes epithelial-mesenchymal transition by inducing senescence escape in endometriosis
Source: Sci Rep. 2022 Jul 19;12:12302. doi: 10.1038/s41598-022-16629-x (PMC9296487; doi:10.1038/s41598-022-16629-x)

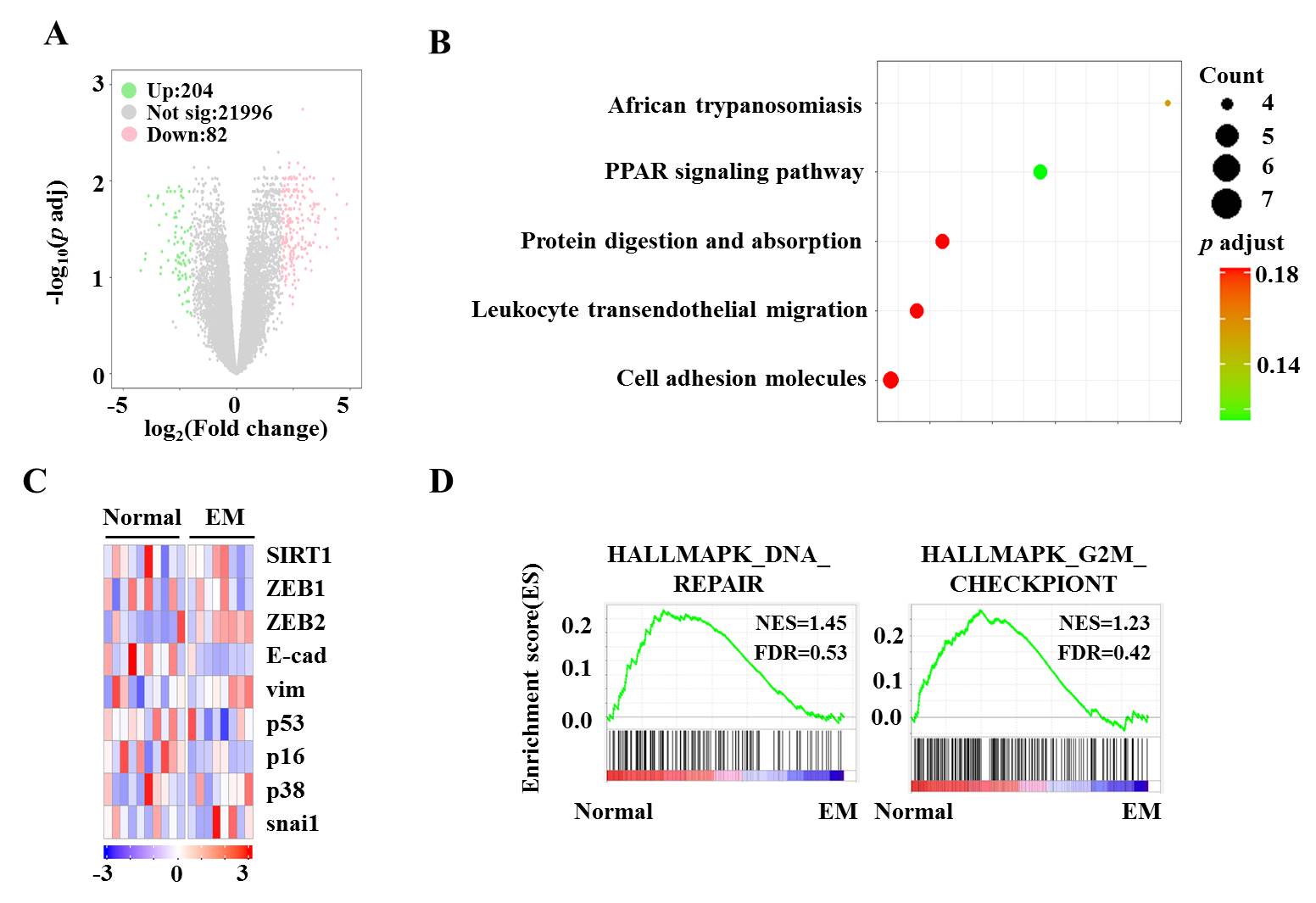

Supplement: Supplementary file 2 — Supplementary Information 2. [file 41598_2022_16629_MOESM2_ESM.jpg]

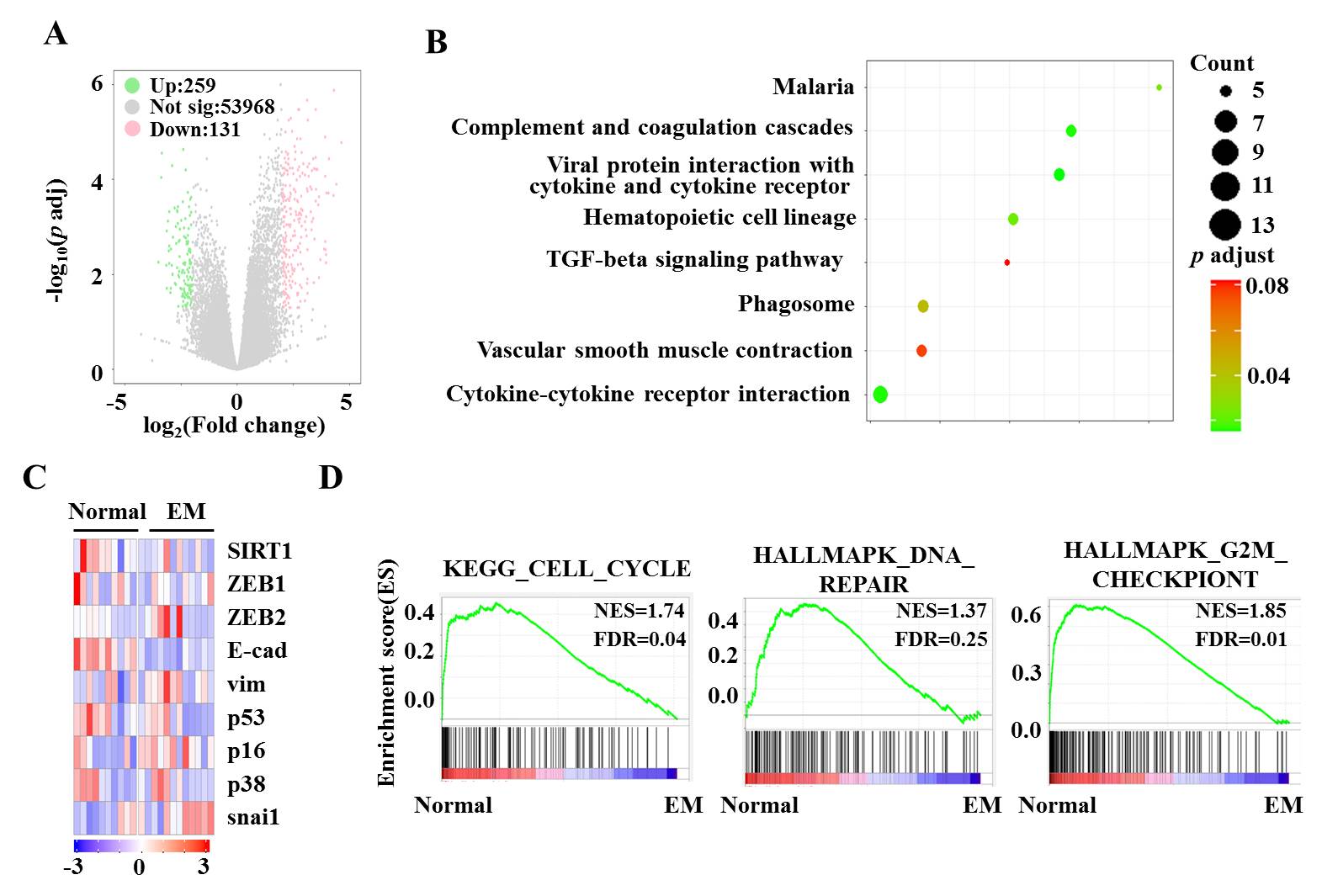

Supplement: Supplementary file 3 — Supplementary Information 3. [file 41598_2022_16629_MOESM3_ESM.jpg]

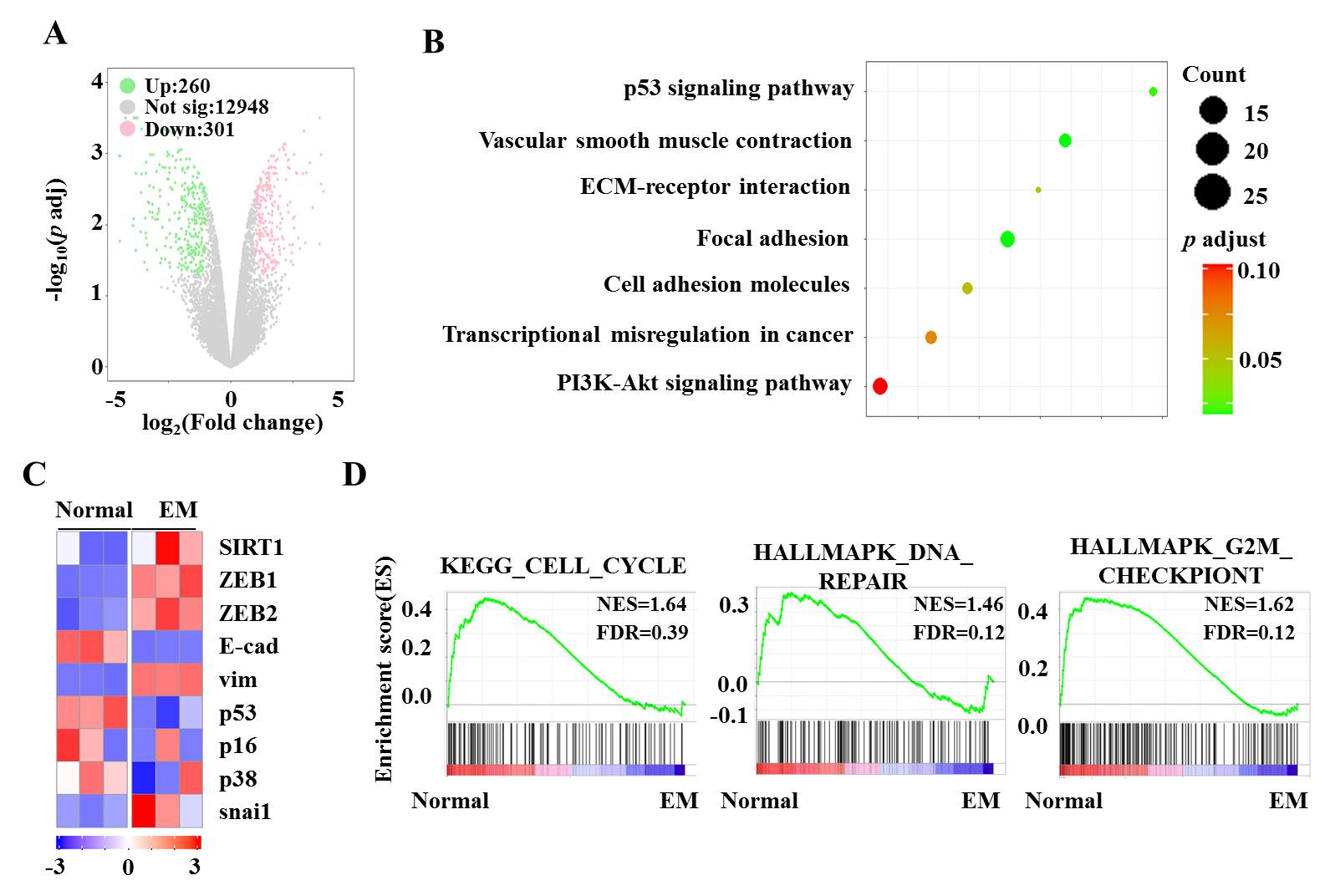

Supplement: Supplementary file 4 — Supplementary Information 4. [file 41598_2022_16629_MOESM4_ESM.jpg]

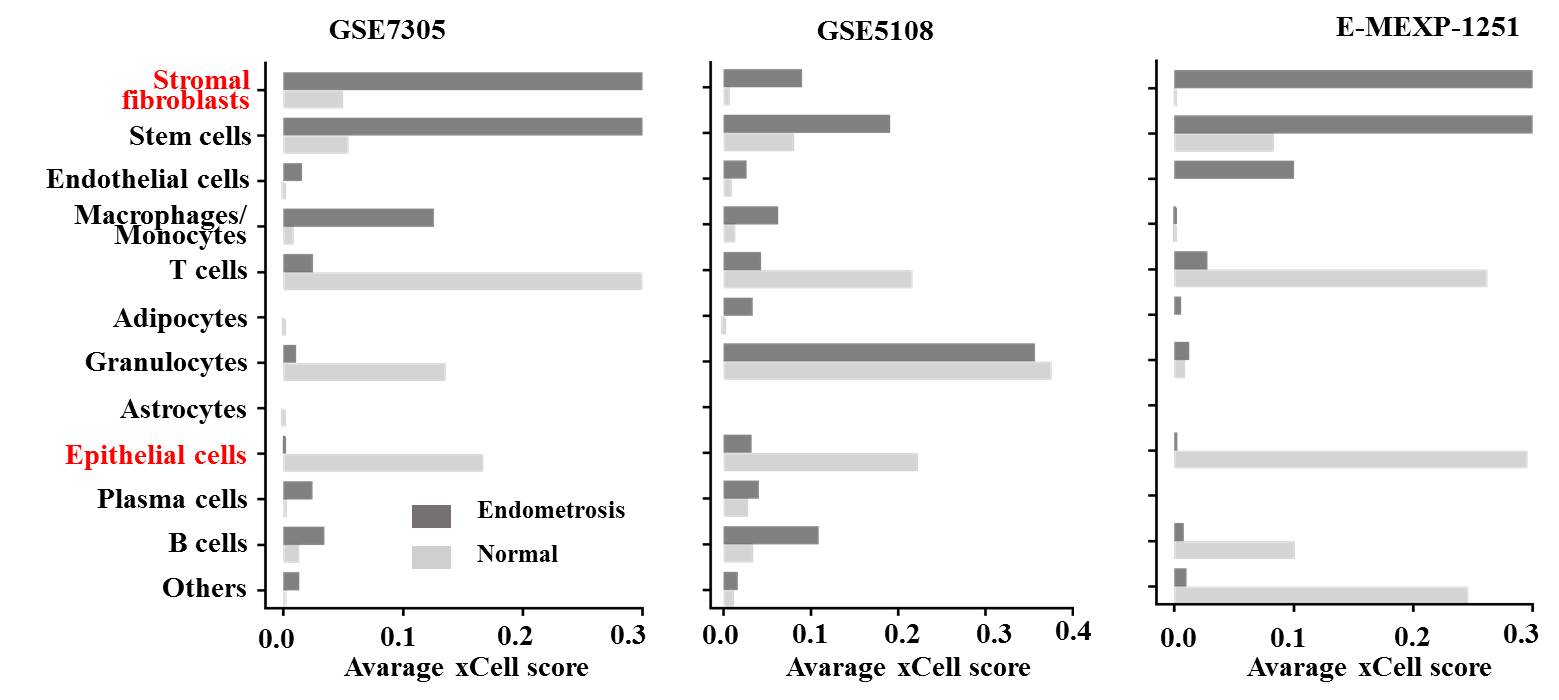

Supplement: Supplementary file 5 — Supplementary Information 5. [file 41598_2022_16629_MOESM5_ESM.jpg]

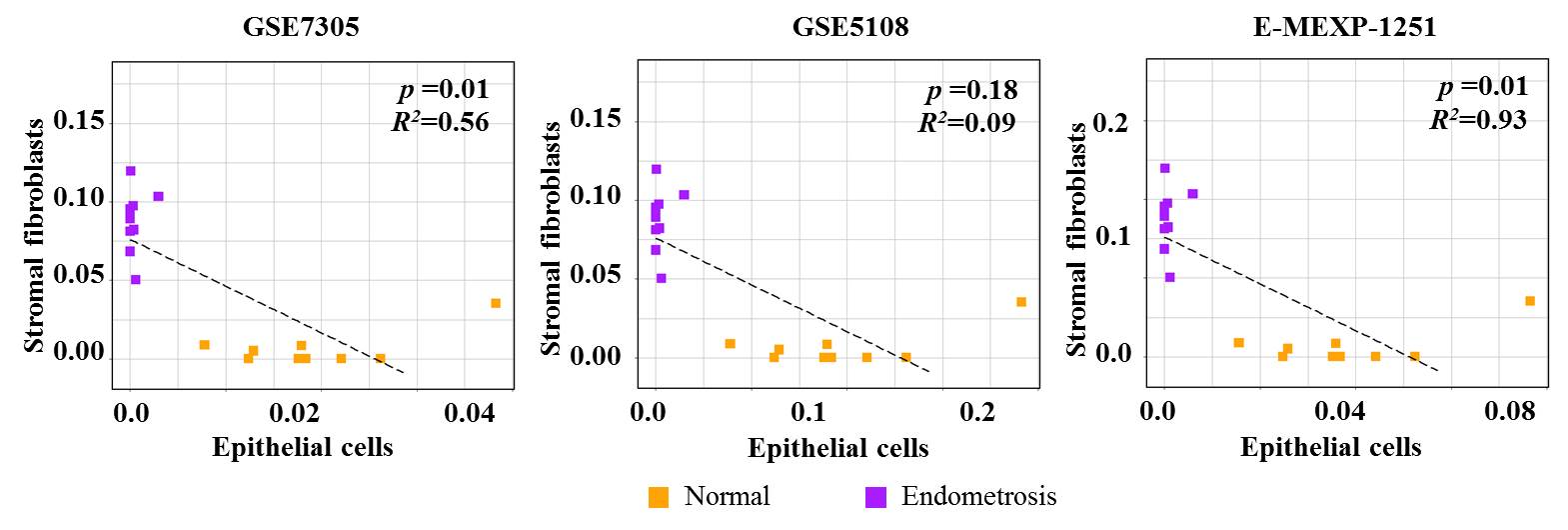

Supplement: Supplementary file 6 — Supplementary Information 6. [file 41598_2022_16629_MOESM6_ESM.jpg]

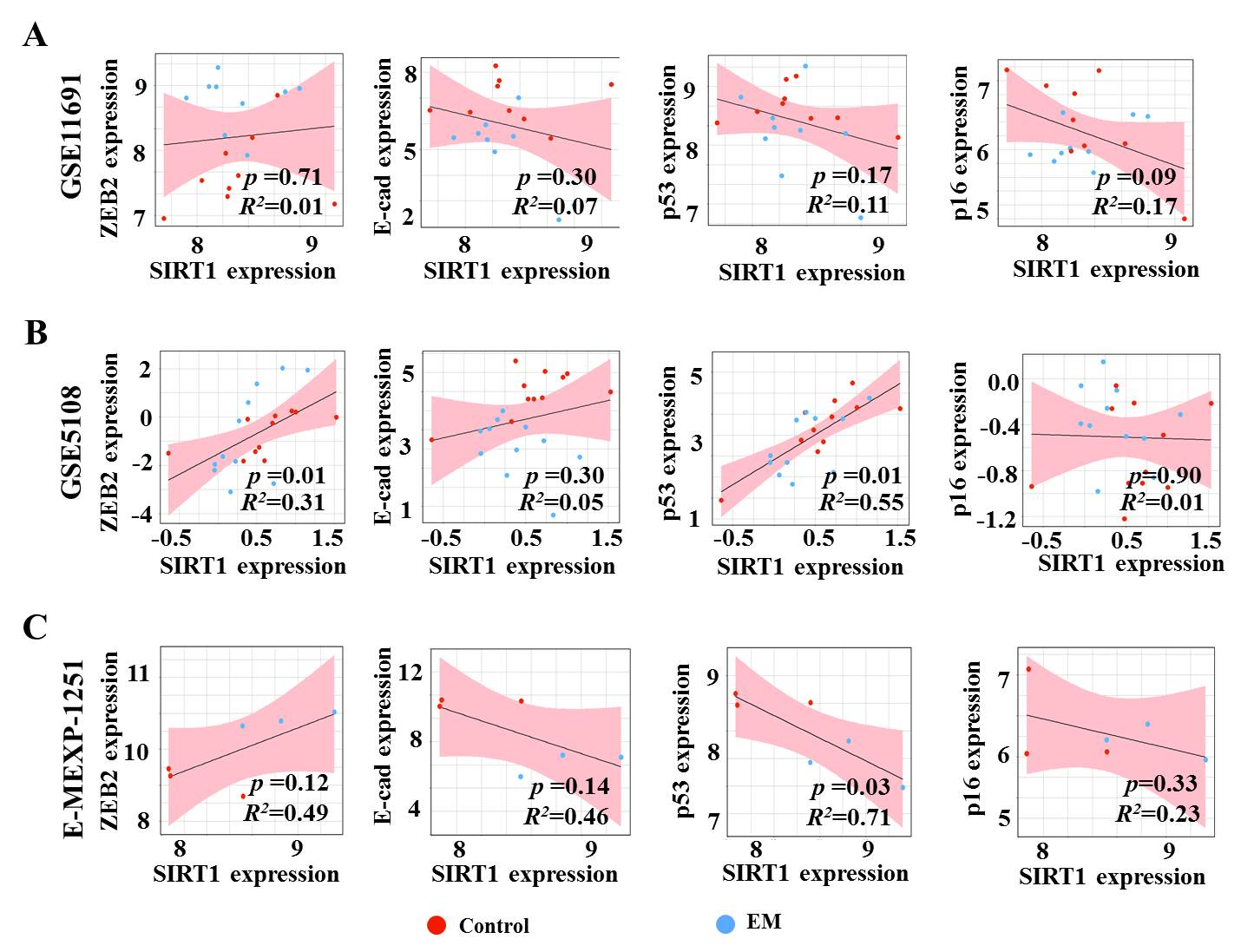

Supplement: Supplementary file 7 — Supplementary Information 7. [file 41598_2022_16629_MOESM7_ESM.jpg]

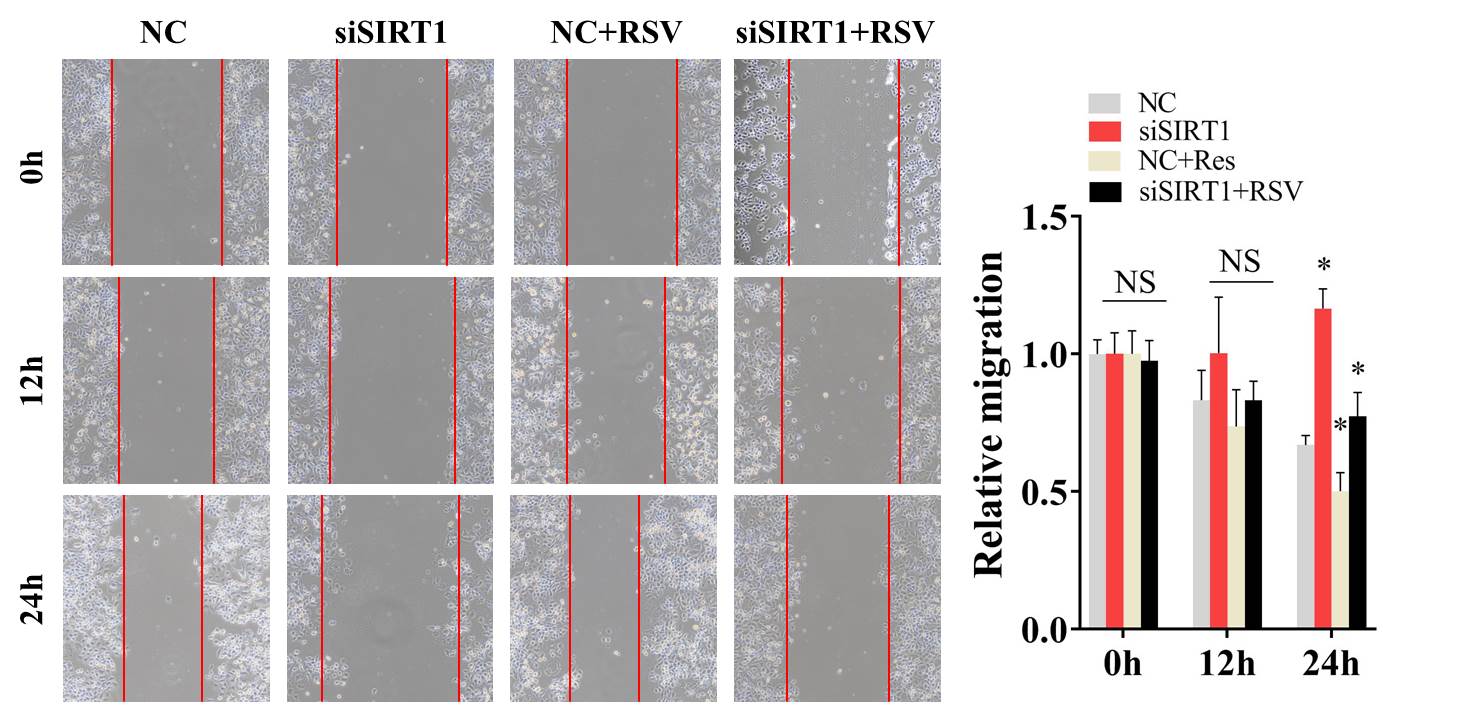

Supplement: Supplementary file 8 — Supplementary Information 8. [file 41598_2022_16629_MOESM8_ESM.jpg]
