# Supplementary material for: SIRT1 upregulation promotes epithelial-mesenchymal transition by inducing senescence escape in endometriosis
Source: Sci Rep. 2022 Jul 19;12:12302. doi: 10.1038/s41598-022-16629-x (PMC9296487; doi:10.1038/s41598-022-16629-x)
Supplement: Supplementary file 10 — Supplementary Information 10. [file 41598_2022_16629_MOESM10_ESM.pdf]

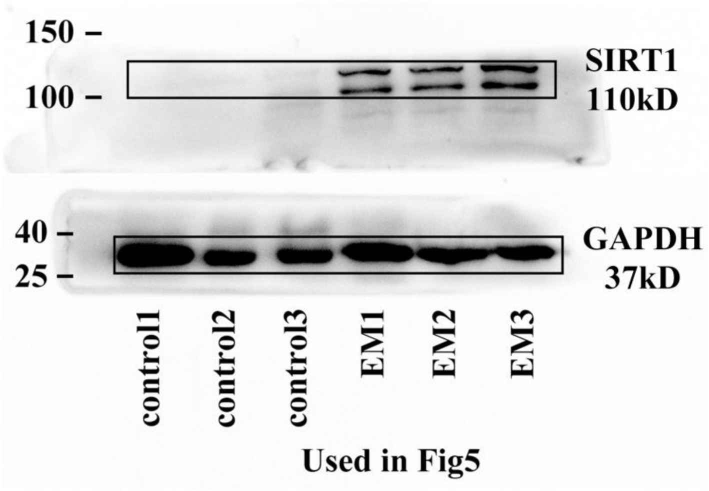

Supplementary Figure S2. Original blots in Fig5.

The gel images and cropped area for western blots of SIRT1 and GAPDH in Fig5F.
