# Supplementary material for: SIRT1 upregulation promotes epithelial-mesenchymal transition by inducing senescence escape in endometriosis
Source: Sci Rep. 2022 Jul 19;12:12302. doi: 10.1038/s41598-022-16629-x (PMC9296487; doi:10.1038/s41598-022-16629-x)
Supplement: Supplementary file 11 — Supplementary Information 11. [file 41598_2022_16629_MOESM11_ESM.pdf]

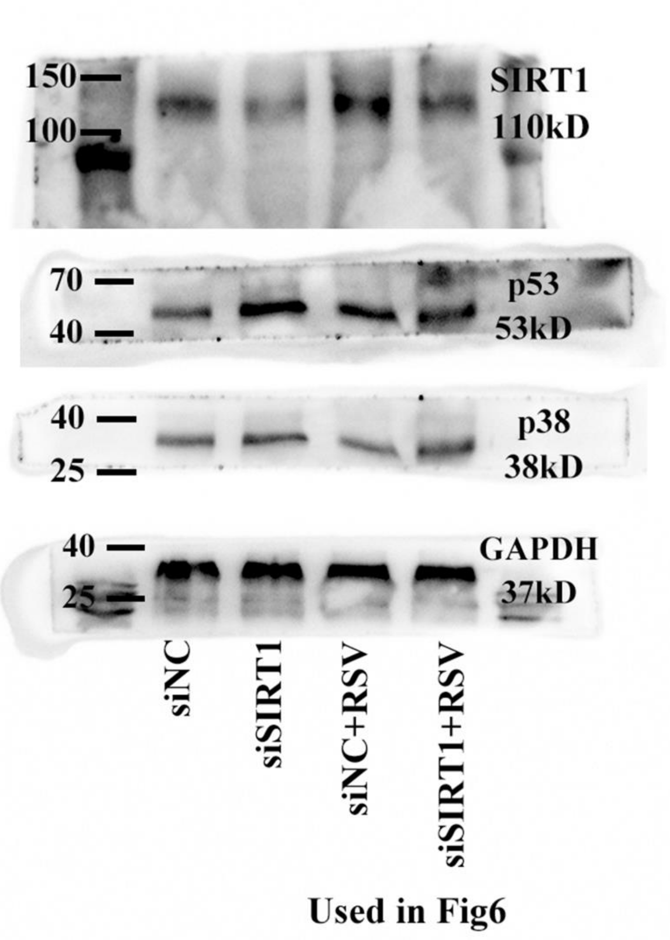

Supplementary Figure S3. Original blots in Fig6D.

The gel images and cropped area for western blots of SIRT1, p53, p38 and GAPDH in Fig6D. p38 bands was stripped by treatment with WB stripping Solution and reproved with GAPDH antibody for confirmation of equivalence of the loading protein in each lane.
