# Supplementary material for: SIRT1 upregulation promotes epithelial-mesenchymal transition by inducing senescence escape in endometriosis
Source: Sci Rep. 2022 Jul 19;12:12302. doi: 10.1038/s41598-022-16629-x (PMC9296487; doi:10.1038/s41598-022-16629-x)
Supplement: Supplementary file 12 — Supplementary Information 12. [file 41598_2022_16629_MOESM12_ESM.pdf]

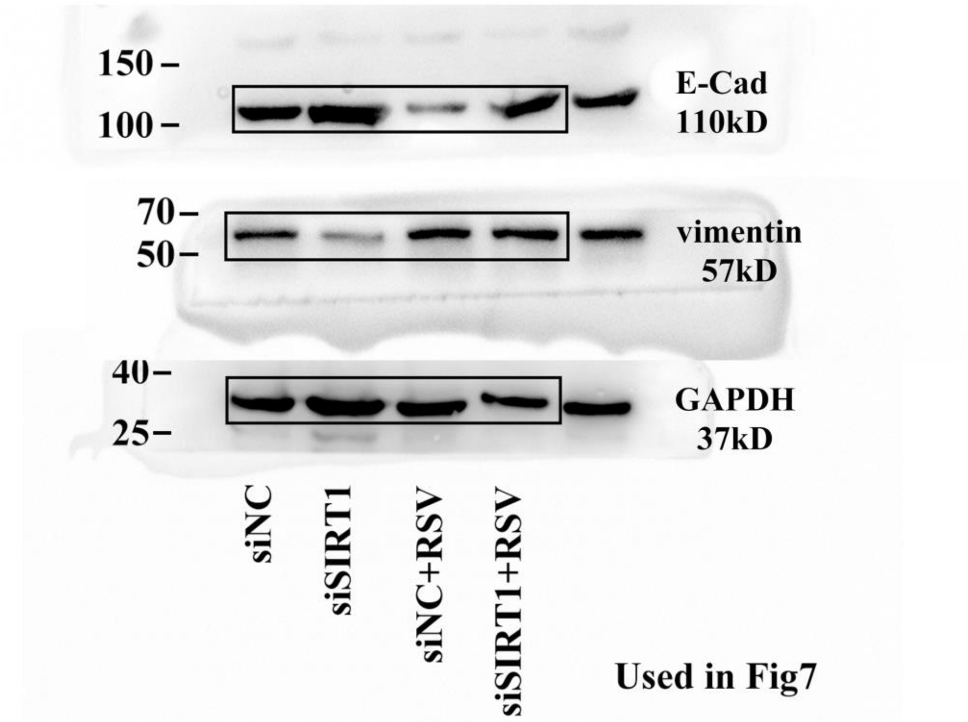

Supplementary Figure S4. Original blots in Fig7C.

The gel images and cropped area for western blots of E-cad, vimentin and GAPDH in Fig7C.
