# Supplementary material for: SIRT1 upregulation promotes epithelial-mesenchymal transition by inducing senescence escape in endometriosis
Source: Sci Rep. 2022 Jul 19;12:12302. doi: 10.1038/s41598-022-16629-x (PMC9296487; doi:10.1038/s41598-022-16629-x)
Supplement: Supplementary file 14 — Supplementary Information 14. [file 41598_2022_16629_MOESM14_ESM.docx]

**Table S2. Primer sets used for RT-PCR, and sequence of siRNAs used for small interference RNA experiments.**

| HUMAN-SIRT1 | Forward:5′-AAGTTGACTGTGAAGCTGTACG-3′;  Reverse:5′-TGCTACTGGTCTTACTTTGAGGG-3′ |
| --- | --- |
| HUMAN-E-cad | Forward: 5′-CCGAGAGCTACACGTTCAC-3′;  Reverse: 5′-AATAGGCTGTCCTTTGTCGAC-3′; |
| HUMAN-vimentin | Forward:5′-AGAGGAAGCCGAAAACACC-3′;  Reverse:5′-AAGATTCCACTTTGCGTTCAAG-3′ |
| HUMAN-P53 | Forward:5′- GAGGTTGGCTCTGACTGTACC-3′;  Reverse:5′- TCCGTCCCAGTAGATTACCAC-3′ |
| HUMAN-p16 | Forward:5′- CGCTTCGATTCTCCGGAAA-3′;  Reverse:5′- CATTCGAGAGATCTGTACGCG-3′ |
| HUMAN-p38 | Forward:5′- CCCGTCTTGGCTTATCCACT-3′;  Reverse:5′- TACATACTGCCGCAGGTCAC-3′ |
| HUMAN-ZEB1 | Forward:5′- AAGTGGCGGTAGATGGTAATGT-3′;  Reverse:5′- AAGGAAGACTGATGGCTGAAAT-3′ |
| HUMAN-ZEB2 | Forward:5′- GGAGACGAGTCCAGCTAGTGT-3′;  Reverse:5′- CCACTCCACCCTCCCTTATTTC-3′ |
| HUMAN-GAPDH | Forward:5′-GGAGCGAGATCCCTCCAAAAT-3′;  Reverse:5′-GGCTGTTGTCATACTTCTCATGG-3′ |
| si-h-SIRT1_001 | 5′-CCAAGCAGCTAAGAGTAAT-3′ |
| si-h-SIRT1_002 | 5′-GCACTCGGTTGTCTTTACT-3′ |
| si-h-SIRT1_003 | 5′-ACTTTGCTGTAACCCTGTA-3′ |
