# Supplementary material for: SIRT1 upregulation promotes epithelial-mesenchymal transition by inducing senescence escape in endometriosis
Source: Sci Rep. 2022 Jul 19;12:12302. doi: 10.1038/s41598-022-16629-x (PMC9296487; doi:10.1038/s41598-022-16629-x)
Supplement: Supplementary file 15 — Supplementary Information 15. [file 41598_2022_16629_MOESM15_ESM.docx]

**Supplementary Table S3.** List of antibodies used in Western blotting.

| Antibody | Art. No. | Dilution | Company |
| --- | --- | --- | --- |
| SIRT1 | ab110304 | 1：1000 | Abcam,USA |
| P53 | 2524 | 1：1000 | CST,USA |
| P16 | ab51243 | 1：1000 | Abcam,USA |
| P38 | 9212 | 1：1000 | CST,USA |
| E-cad | 610181 | 1：1000 | BD,USA |
| Vimentin | 5741 | 1：1000 | CST,USA |
| GAPDH | 97166 | 1：1000 | CST,USA |
